# OligArch: A software tool to allow artificially expanded genetic information systems (AEGIS) to guide the autonomous self-assembly of long DNA constructs from multiple DNA single strands

**DOI:** 10.3762/bjoc.10.192

**Published:** 2014-08-11

**Authors:** Kevin M Bradley, Steven A Benner

**Affiliations:** 1 Foundation for Applied Molecular Evolution, P.O. Box 13174, Gainesville FL 32604, USA; 2 The Westheimer Institute for Science and Technology, 720 S. W. 2nd Avenue, Suites 201-208, Gainesville FL 32601, USA; 3 Firebird Biomolecular Sciences LLC, 13709 Progress Blvd. Box 17, Alachua, FL 32615, USA

**Keywords:** AEGIS, bioinformatics, DNA self-assembly, long DNA constructs, software

## Abstract

Synthetic biologists wishing to self-assemble large DNA (L-DNA) constructs from small DNA fragments made by automated synthesis need fragments that hybridize predictably. Such predictability is difficult to obtain with nucleotides built from just the four standard nucleotides. Natural DNA's peculiar combination of strong and weak G:C and A:T pairs, the context-dependence of the strengths of those pairs, unimolecular strand folding that competes with desired interstrand hybridization, and non-Watson–Crick interactions available to standard DNA, all contribute to this unpredictability. In principle, adding extra nucleotides to the genetic alphabet can improve the predictability and reliability of autonomous DNA self-assembly, simply by increasing the information density of oligonucleotide sequences. These extra nucleotides are now available as parts of artificially expanded genetic information systems (AEGIS), and tools are now available to generate entirely standard DNA from AEGIS DNA during PCR amplification. Here, we describe the OligArch (for "oligonucleotide architecting") software, an application that permits synthetic biologists to engineer optimally self-assembling DNA constructs from both six- and eight-letter AEGIS alphabets. This software has been used to design oligonucleotides that self-assemble to form complete genes from 20 or more single-stranded synthetic oligonucleotides. OligArch is therefore a key element of a scalable and integrated infrastructure for the rapid and designed engineering of biology.

## Introduction

Automated synthesis of single stranded DNA fragments has, perhaps more than any other technology, enabled the development of "synthetic biology“ as a modern field over the past 30 years [1–5]. While oligonucleotides can be reliably prepared by automated synthesis up to ca. 100 nucleotides in length and (even today) are most often used as primers, many seek to create large DNA (L-DNA) constructs by assembly of these fragments. Such engineered L-DNA might encode new and useful functions, including the manufacturing of biofuels, the synthesis of pharmaceuticals, and the development of new materials.

As it is taught to non-chemists, DNA appears to be an ideal molecule for such “hands-off” self-assembly. In this idealized "cartoon“, two strands of DNA bind to each other perfectly, so long as their sequences are arranged so that A pairs with T and G pairs with C.

Even funding agencies have been captivated by this vision. For example, in 2011 the Army Research Office issued a small business grant solicitation seeking companies to design software to design 30,000 base pairs of single stranded DNA that would autonomously self-assemble to form nanostructures. In 2012, DARPA issued a small business grant solicitation seeking technology to assemble single-stranded synthetic fragments to give 20,000 base pair DNA constructs, essentially under this simple model for DNA behavior. More recently, DARPA has initiated its "Foundries 1000“ program, where large DNA "chassis" are hoped to occur in an entirely automated process.

If DNA in fact behaved according to this ideal, then the specificity of Watson–Crick nucleobase pairing might indeed allow autonomous self-assembly of an unlimited number of DNA strands to give L-DNA constructs of indefinitely large lengths. All that would be necessary is to design the requisite number of synthetic single strand fragments to remove off-target annealing, make them, and mix them. Once mixed, the designed strands would, in this view, simply fall together to form the target L-DNA structure.

**Figure 1:**
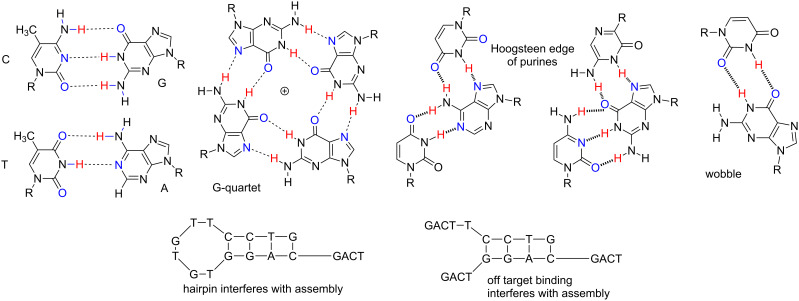
Some structures showing possible reasons why large DNA (L-DNA) constructs do not self-assemble from more than approximately a dozen synthetic single-stranded DNA oligonucleotides. Top from left to right: The presence of strong (C:G) and weak (T:A) nucleobase pairs complicates the design of self-assembling fragments. G-quartets can arise from G-rich sequences, with major groove interactions involving hydrogen bonding to the “Hoogsteen edge” of purines. Wobble pairing can compete with Watson–Crickery. Bottom. Even if Watson–Crickery were the only way for single stranded DNA sequences to interact, the low information density of four-nucleotide DNA allows easy off-target hybridization and unimolecular hairpin formation. Unimolecular processes (such as hairpin formation) compete with the desired intermolecular hybridization, especially at low concentrations of oligonucleotide.

Unfortunately, this simple model is also simplistic. With just four nucleotides, the information density of standard DNA is too low to allow (without exquisite design) even a dozen single strands to reliably self-assemble upon simple mixing. Further, even if rule-based Watson–Crick pairing were to be the only possible interaction, the combination of “strong” and “weak” G:C and A:T pairs makes design challenging. Also able to defeat self-assembly, DNA molecules can easily fold to give single-strand structures (such as hairpins), this unimolecular process competes with intermolecular duplex formation. Finally, a rich repertoire of non-Watson–Crick interactions (e.g., wobble, major groove binding) can compete with Watson–Crickery (Figure 1) to render autonomous self-assembly impossible.

Even with great advances in recent years in large-scale DNA assembly using natural bases, the chance of failure of these assemblies grows very high once the number of fragments increases. With our own design that used fragments whose sequences were optimized for self-assembly [6], autonomous assembly typically failed between 16 and 24 oligonucleotides. Other methods of assembly, such as the Gibson Assembly [7] or SLIC [8], either limit the number of fragments to be used or rely on stepwise assembly of such syntheses, with the recommendation from Gibson that autonomous self-assembly of single stranded DNA fragments “be limited to perhaps a dozen fragments at a time”.

**Figure 2:**
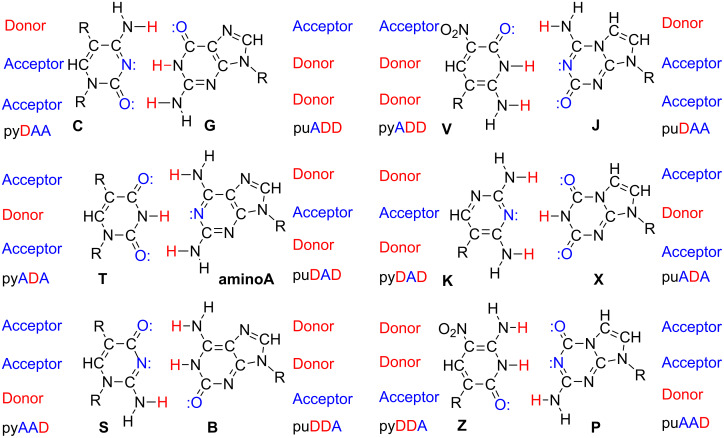
Watson–Crick pairing rules follow two rules of complementarity: (a) size complementarity (large purines pair with small pyrimidines) and (b) hydrogen bonding complementarity (hydrogen bond acceptors, A, pair with hydrogen bond donors D). Rearranging donor and acceptor groups on the nucleobases creates an artificially expanded genetic information system (AEGIS), whose components can independently pair. AEGIS adds information density to the DNA oligonucleotides, thereby diminishing off-target hybridization and other undesired aggregation/folding motifs. With strength comparable to the G:C pair, AEGIS components form **S**:**B**, **Z**:**P**, **V**:**J**, and **K**:**X** pairs.

Fortunately, another development of synthetic biology offers an approach to mitigate these limitations of natural DNA as a matrix for autonomous self-assembly. This exploits a "second-generation" version of an artificially expanded genetic information system (AEGIS) [3,9]. AEGIS adds nucleotide building blocks to the four found in standard DNA (G, A, C, and T) by shuffling hydrogen-bonding units on the nucleobases, all while retaining the overall Watson–Crick nucleobase pairing geometry (Figure 2). These extra nucleotides bind to form additional nucleobase pairs orthogonally to the A:T and G:C pairs.

In principle, adding extra nucleotides in the genetic alphabet can mitigate the hybridization problems in highly complex mixtures of single-strand DNA fragments simply by increasing the information density of the resulting DNA sequences. With four nucleotides, the number of possible 15mers (which form duplexes with convenient melting temperatures) is approximately 1.1 billion (≈ 4^15^). While this number might appear to be large, it includes an enormous range of melting temperatures, a range arising because of the relative strengths of the G:C and A:T pair. Adding two additional nucleotides increases the number of potential hybridizing 15mers to 470 billion (≈ 6^15^), nearly 500 fold higher. Adding four AEGIS nucleotides increases this number to 35 trillion (≈ 8^15^). With a full AEGIS alphabet containing 12 nucleotide letters, approximately 1.5 quadrillion 15mers (1.54 × 10^16^ ≈ 12^15^) are conceivable to serve as orthogonal hybridizing units. Further, AEGIS pairs are joined by three hydrogen bonds, giving them the strength of C:G pairs [3]. By increasing the information density of DNA, AEGIS DNA should more easily support “no hands” self-assembly by greatly increasing the number of possible unique fragments and maximize uniqueness of fragment ends.

Of course, a large DNA construct built with AEGIS nucleobases would, after it is assembled, still contain AEGIS components. For those who want an entirely natural end product, this is undesirable. Therefore, tools are needed to replace AEGIS pairs by standard pairs in processes that are rigorously rule-based.

**Figure 3:**
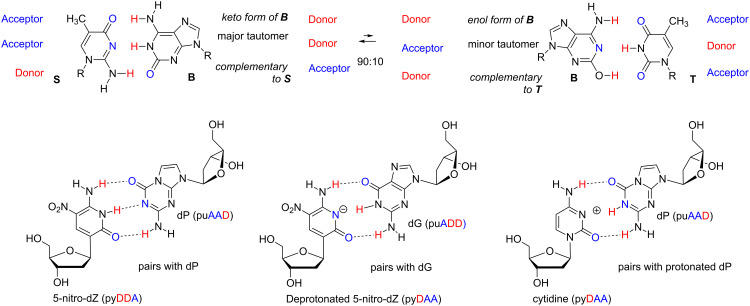
(top) The conversion of **S**:**B** pairs to T:A pairs involves tautomerization of **B** to give its minor enol form, which present a hydrogen bond Donor–Acceptor–Donor pattern complementary to T. If a strand containing **B** is copied by a polymerase that is not given any d**S**TP, mismatching of T opposite a minor enol tautomer of **B** leads (after two cycles of copying) to the replacements of **S**:**B** pairs by T:A pairs. (bottom) The conversion of **Z**:**P** pairs to C:G pairs involves the mismatching of C opposite a protonated **P**, and/or the mismatch of deprotonated **Z** opposite G. Thus, if a strand containing **Z** is copied at high pH by a polymerase that is not given any d**P**TP, mismatching of G opposite deprotonated **Z** leads (after two cycles of copying) to the replacements of **Z**:**P** pairs by C:G pairs. Conversely, if a strand containing **P** is copied at low pH by a polymerase that is not given any d**Z**TP, mismatching of G opposite protonated **P** leads (after two cycles of copying) to the replacements of **Z**:**P** pairs by C:G pairs.

Conversion of AEGIS nucleotides to standard nucleotides, it turns out, is facile by four AEGIS components: 2’-deoxy-5-methylisocytidine (trivially named **S**), 2’-deoxyisoguanosine (trivially named **B**), 2-amino-8-(1’-β-D-2’-deoxyribofuranosyl)-imidazo[1,2-*a*]-1,3,5-triazin-4(8*H*)one (trivially named **P**), and 6-amino-5-nitro-3-(1’-β-D-2’-deoxyribofuranosyl)-2(1*H*)-pyridone (trivially named **Z**) (Figure 2). In both cases, conversion is facilitated by forcing polymerases to mismatch AEGIS nucleotides with standard nucleotides by depriving the polymerase of the complementary AEGIS triphosphate. The specificity of mismatching is driven by intrinsic features of the AEGIS nucleobase. Thus, the **B**:T mismatch is enabled by a minor tautomeric form of **B**. The **Z**:G mismatch is enabled by the deprotonation of **Z**. The **P**:C mismatch is enabled by the protonation of **P** (Figure 3). These mismatches can occur both in vitro using PCR [10] and in vivo, in an engineered strand of *E. coli*. While rules for conversion have complexities, in their simplest forms, the **S**:**B** and **Z**:**P** pairs are converted to C:G and T:A pairs, respectively.

The availability of this new concept for the assembly of large DNA constructs from multiple inexpensive single-stranded oligonucleotides, together with the chemistry and enzymology needed to convert unnatural assemblies into entirely natural assemblies, creates the need for a software product to assist in the design of the fragments to be assembled. That product, OligArch (for “oligonucleotide architecting”) is described here. While other packages exist that output designed oligonucleotides for large-scale synthesis, such as GeneGenie [11], DNAWorks [12], and Gene2Oligo [13], these all work with only natural bases, and require either a limited number of DNA fragments or a multistep process. OligArch is unique in allowing the use of AEGIS bases to greatly expand the number of fragments that can be used in a single-step self-assembly.

## Discussion

### Brief summary of OligArch software package

As input, the OligArch software package takes a sequence for a desired target DNA construct. As output, OligArch delivers sequences for a set of oligonucleotide fragments that include components of an artificially expanded genetic information system (AEGIS). OligArch designs these fragments so that the target DNA is produced after the fragments are annealed, after the annealed fragments are (optionally) extended by a DNA polymerase to fill in any gaps to give nicked DNA, after any nicks are sealed, and after the AEGIS pairs are replaced by standard pairs. OligArch also ensures that the increased information density of AEGIS oligonucleotides is exploited to avoid hairpin formation, off-target annealing, and undesired non-canonical structures. Thus, OligArch is a tool critical for exploiting the extra information density in AEGIS alphabets to assemble large DNA molecules.

The user of OligArch can designate within a given target DNA construct specific regions that encode proteins; OligArch ensures that the expressed protein is unchanged by the reassembled sequence. Further, the user can enter “sequence-dependent” regions (such as promoters, replication origins, etc.), and the application will ensure that the nucleotide sequence is completely unchanged in the reassembled sequence. Finally, short “non-changeable” regions (such as restriction enzyme recognition sites) can be entered to ensure no AEGIS substitutions occur at these locations.

### User interface

Using OligArch’s web-based form, the user enters the sequence to be designed and specifies if the program should create oligonucleotides with short overlapping AEGIS spans (requiring extension by polymerase) or oligonucleotides with fully overlapping sequences. Also entered is the location of protein-coding, sequence-dependent, and non-changeable regions. The user then chooses design criteria such as optimal oligonucleotide length, longest and shortest acceptable oligonucleotides, number of AEGIS bases to use within AEGIS spans, AEGIS span melting temperatures (*T*_m_), and other parameters.

Also customizable are criteria used for *T*_m_ calculations, including concentrations of oligonucleotides, Na^+^, and Mg^2+^. The user may choose to use the AEGIS pairs **Z**:**P**, **S**:**B**, or **Z**:**P**/**S**:**B** for substitutions. Finally, the user can choose whether or not the designed sequence is circularized and whether or not oligos should be divided into individual assembly sets to allow step-wise assembly (only required for very large targets). Once the above information is submitted, OligArch runs using the algorithm described below. The designed oligonucleotides, along with any necessary warnings, are then displayed to the user in a table. Further, a detailed file can be downloaded with the designed oligonucleotides aligned to the original sequence in both tabular and graphical format.

### Algorithm

OligArch attempts to create easily synthesizable oligos utilizing AEGIS base technology to ensure unique recognition sites exist in overlapping oligos. Once a user has input the sequence to be synthesized, sequence regions, and other design criteria, OligArch scans the sequence looking for positions where natural bases (A, G, T or C) could be substituted with AEGIS bases. These potential substitutions are stored along with the original bases in an indexed array. In designating potential substitutions, each of three categories of sequence regions have distinct rules:

1. Protein-coding regions: AEGIS nucleobases can be substituted only at the third site in codons where any nucleobase in the third position produces the same amino acid (Leu, Val, Ser, Pro, Thr, Ala, Arg, and Gly), as well as codons where C/T or A/G are interchangeable in the third position (His, Gln, Asn, Lys, Asp, and Glu). The following AEGIS substitutions are allowed: **P** for a G, **Z** for a C, **B** for an A, and **S** for a T.
2. Sequence-dependent regions: AEGIS nucleobase substitutions in these regions are only allowed where known rules allow conversion back to ACTG bases results in the exact same sequence. Two such rules exemplify this type of substitution for **Z**:**P** pairs, which are **PP** to GG (and conversely **ZZ** to CC) and **P**T**P** to GTG (and conversely **Z**A**Z** to CAC) (Shaw & Benner, unpublished). For **S**:**B** pairs, any **S**:**B** pairs that are flanked on either side by a natural base, N, have 100% conversion back to T:A respectively (NSN to NTN and NBN to NAN)
3. Non-critical regions *(*default*)*: Regions that are not critical to the sequence allow AEGIS substitution at any base. As with protein-coding regions, the following AEGIS substitutions are allowed: **P** for a G, **Z** for a C, **B** for an A, and **S** for a T.

The forth category of sequence region, non-changeable regions, does not allow an AEGIS base substitution and is limited to a maximum size of 12 nucleotides.

Once this sequence substitution array has been created, OligArch searches for oligonucleotides that meet the user-entered specifications. Each oligonucleotide is composed of “AEGIS spans” on both ends, with optional AGTC sequence between each span. The AEGIS span is a sequence of nucleobases that contains AEGIS substitutions and that uniquely overlaps with its complement span. AEGIS spans attempt to have the optimal number of AEGIS–nucleobase substitutions, and must contain the minimal number of substitutions.

For short overlaps, the default size of the AEGIS spans is 12 to 15 nts with a required *T*_m_ between 46 and 58 °C, and the minimum number of AEGIS substitutions is two per span (with optimal set at four). For complete overlaps, the AEGIS spans cover half of the designed oligonucleotides, with a required *T*_m_ of 70 °C or greater, and a minimum of four AEGIS substitutions (with optimal set at 6). *T*_m_’s are calculated using the nearest-neighbor method along with unified entropy and enthalpy values from SantaLucia, et al. [14]. AEGIS bases **B** and **P**, both purines able to make 3 hydrogen bonds, are treated as G within *T*_m_ calculations, while **S** and **Z**, both pyrimidines able to form 3 hydrogen bonds, are treated as C. The exact *T*_m_ formula is:


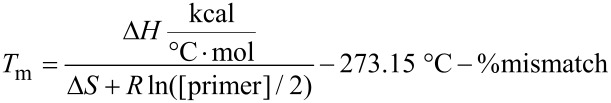


where *R* is the universal gas constant (1.987 cal/°C·mol) and Δ*H* and Δ*S* are the enthalpy and entropy of the base stacking adjusted for initiation factors [14], with Δ*S* also adjusted for salt concentrations using the formula [15]:


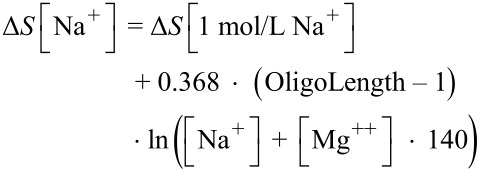


AEGIS segments are designed using a bidirectional sliding-window approach. Starting at the first position in the sequence, a sequence substitution array is used to design all possible AEGIS spans within the user-specified size range that match the input design criteria. AEGIS spans are then examined to include only those with an AEGIS nucleobase located within a user-specified number of bases from both the 5’- and 3’-end (the default is four nucleotides) to minimize unwanted complementarity. Remaining spans are then ranked based upon number of AEGIS nucleobases, distribution of AEGIS nucleobases, and presence and size of repeating nucleotide patterns.

Once ranked, these spans are compared against all other AEGIS spans being used in the current assembly set, with those that might hybridize with an unintended target being excluded. Potential hybridization is defined as any complementarity with a *T*_m_ of ≥50% of the minimum span *T*_m_ that can be designed. Further, spans that will result in hairpin formation within designed primers are excluded. Hairpin formation is checked using the algorithm found in Primer3 [16–17] with a required max self-complementary score of 4 and additionally requiring a *T*_m_ of at least 6 °C in the stem sequence. Span comparisons continue until a single span is found that meets the above criteria. If no span can be created within the optimal window, the window is moved until an acceptable span can be created. With the bidirectional approach, the window moves 1 nt upstream from the optimal position, followed by 1 nt downstream, 2 nt upstream, 2 nt downstream, etc. until a valid AEGIS span can be found.

OligArch then attempts to design the next AEGIS span, which will be located downstream of the first span, in such a location that the two AEGIS spans (plus natural base sequence between the spans, if using short overlaps) creates an oligonucleotide of optimal length. The two AEGIS spans and any sequence between the spans become the first oligonucleotide, with the complement of the 2^nd^ span becoming the start of the second nucleotide. This process continues until the entire sequence has been designed as individual nucleotides. On rare occasions, OligArch may need to design an oligonucleotide that is longer or shorter than the user-specified criteria; a warning is issued if this occurs. For complete overlaps without gaps to fill, OligArch will also attempt to redesign the leading oligonucleotide, if necessary to allow proper design.

For circularized sequences, the complement of the first and last AEGIS span is used to create the final oligonucleotide sequences which will hybridize to circularize the product. If necessary, the program adjusts the lengths of the flanking fragments to ensure the final oligonucleotide is on the proper strand. For linear sequence, any sequence at the 5’- or 3’-end that could not be included in an AEGIS span is added to the end of the terminal oligonucleotides.

### Results of OligArch designed assembly

**Figure 4:**
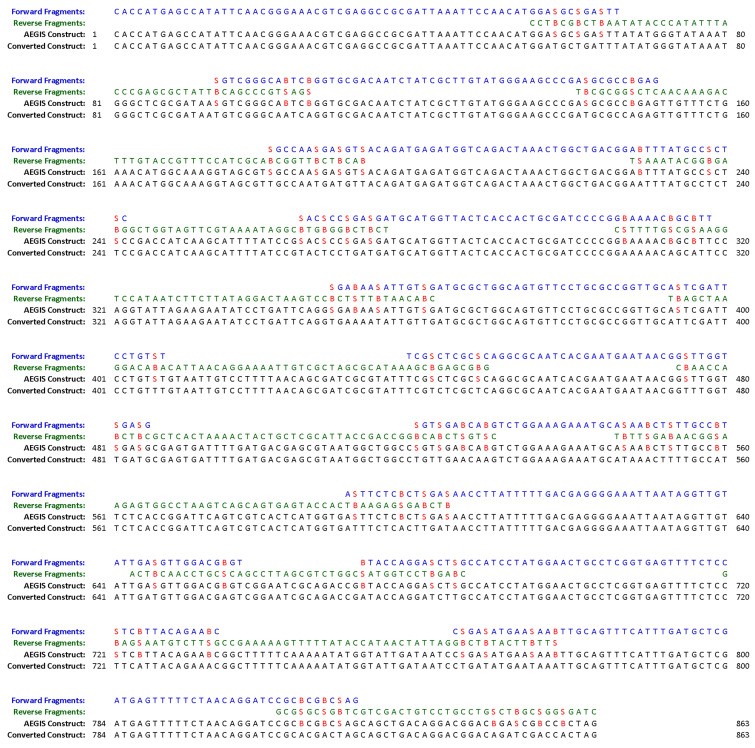
Representative assembly of oligonucleotides designed by OligArch built from the components of the six-nucleotide AEGIS GACT**SB** alphabet. The top two lines show the fragments. The line below shows the product before conversion. The bottom line shows the end product, entirely natural DNA, after conversion. This example, from [6], leads to the autonomous self-assembly of a gene that confers resistance to the antibiotic kanamycin.

The combination of the OligArch software with the ability to convert AEGIS nucleotides to standard nucleotides allows AEGIS to support the generation of large DNA constructs using the architecture described above. A representative assembly is shown in Figure 4, taken from the total synthesis of a gene encoding kanamycin resistance created using this strategy [6]. Here, oligonucleotides containing AEGIS components are designed by OligArch, with the protein coding region preserved to ensure the correct amino acid sequence is produced. These oligonucleotides are chemically synthesized, using chemistry entirely analogous to the chemistry used to synthesize standard DNA. The higher information density of the 6-letter DNA is then used to guide the autonomous assembly of large constructs by simple annealing. Gaps, if any, are filled in by polymerase extension, and the nicks in the product are sealed enzymatically with DNA ligase. After the higher information density provided by the AEGIS components has been exploited, the AEGIS components are removed via PCR to leave an entirely natural final DNA product, which was both complete and fully functional [6].

With the successful synthesis of the kanamycin resistance gene, we show that the AEGIS bases can be used for autonomous self-assembly of large-scale sequences, with conversion back to natural bases to preserve function of these sequences. With the theoretical ability to incorporate up to 8 AEGIS bases, we can far exceed the fragment count limits of natural base oligonucleotides inherent in current assembly methods [7–8], while also avoiding the need for step-wise assembly. With the implementation of OligArch, this technology will be easily harnessed for large scale sequence assembly.

### Computer support and access to OligArch

OligArch is web-based application written in VB.net and utilizing ASP.net technology. It is hosted on a Dell R410 server running Windows Server 2008 R2 Standard. It is publicly accessible at http://bioinformatics.ffame.org/bioinformatics/OligArch.aspx.
